# Acute splenic torsion in an adolescent with polysplenia syndrome: case report

**DOI:** 10.1186/s13052-025-02059-8

**Published:** 2025-07-18

**Authors:** Flora Vassallo, Fabio Panzuto, Gregorio Serra, Anna Maria Caruso, Laura Putignano, Denisia Bommarito, Giovanni Corsello, Sergio Salerno

**Affiliations:** 1 Pediatric Radiology Unit, Children’s Hospital “G. Di Cristina”, University of Palermo, Palermo, Italy; 2 Department of Health Promotion, Mother and Child Care, Internal Medicine and Medical Specialties “G. D’Alessandro”, University of Palermo, Palermo, Italy; 3 Pediatric Surgery Unit, Di Cristina Benfratelli National and Specialist Hospital, Palermo, Italy

**Keywords:** Acute abdomen, Splenic infarction, PSS, Left isomerism, Case report

## Abstract

**Background:**

Splenic torsion is a rare but potentially serious event. It can occur in association with various anatomical anomalies, such as polysplenia. The presence of multiple small spleens is often associated with other abdominal and chest abnormalities, defining a condition known as Polysplenia Syndrome (PSS).

**Case presentation:**

We report on a 14-year-old girl who presented with a 3-day history of left lower abdominal pain and vomiting, with a known history of congenital heart disease associated with *Situs Viscerum Inversus* and polysplenia. She was found to have normal vital signs but elevated C-reactive protein, and leukocytosis with neutrophilia. In light of the clinical and laboratory data, in addition to the suggestive imaging findings revealed on US and contrast-enhanced abdominal CT, a splenic torsion was suspected. The patient underwent a laparotomy, which confirmed a pedunculated mass in the left flank and iliac fossa, consistent with torsion of one of the spleens, and allowing complete resection of the lesion. The postoperative course was uneventful, and she was discharged after 10 days.

**Conclusions:**

Splenic torsion in PSS is an extremely rare condition that may present in a time-critical manner, with nonspecific clinical manifestations and abnormal laboratory results, representing a real diagnostic challenge.

## Background

Splenic torsion is a rare but potentially serious condition due to the abnormal rotation of the spleen around its vascular pedicle, leading to torsion of the splenic vessels. This rotation results from the absence or laxity of the splenic suspensory ligaments—primarily the gastrosplenic, splenorenal, phrenicosplenic, and splenocolic ligaments—which normally anchor the spleen in the left upper quadrant. Without proper ligamentous fixation, the spleen becomes hypermobile, increasing the risk of twisting around its elongated vascular pedicle. Torsion compromises venous return first, leading to venous congestion and splenic enlargement. If uncorrected, arterial flow is subsequently reduced or obstructed, resulting in ischemia, infarction and, ultimately, splenic necrosis. The severity of torsion varies, with clinical manifestations ranging from intermittent abdominal symptoms, when the torsion is partial, to a full-blown acute abdomen in complete torsion. This event may be associated with various anatomical anomalies, including polysplenia. This latter is a rare congenital condition characterized by the presence of multiple spleens within the abdominal cavity. It occurs when the embryonic developmental process of the spleen fails to complete correctly, leading to the formation of multiple splenic organs instead of a single one [1, 2]. Polysplenia is often related with other congenital anomalies, such as heart defects (bilateral superior vena cava, interruption of the inferior vena cava with azygos continuation, ventricular septal defect, *ostium primum* defect, and morphologic left ventricular outflow obstruction), vascular abnormalities, and gastrointestinal malformations (right-sided stomach, short pancreas, midline/left-sided liver, malrotation, inferior vena cava anomalies) [3, 4]. Such association defines the Polysplenia syndrome (PSS), which occurs in approximately 1 in 250,000 live births [5, 6]. Polysplenia can present with a variety of manifestations and be diagnosed in either childhood or adulthood [7–9], although the majority of cases recurs in children. The most common clinical manifestations include abdominal pain, nausea, vomiting, recurrent infections, and gastrointestinal disturbances. Early recognition of polysplenia is crucial for optimal management. It requires a multidisciplinary evaluation involving different specialists within the pediatric area, including gastroenterologists, cardiologists, surgeons, and radiologists [10, 11]. Imaging investigations, such as ultrasound (US), computed tomography (CT), and magnetic resonance imaging (MRI), are often used to confirm the diagnosis and assess for any associated complications [12–14] such as splenic torsion [7], which can lead to ischemia and necrosis, requiring emergency surgery [15, 16].

## Case presentation

Our patient is a 14-year-old European girl with a known history of congenital heart disease (partial anomalous pulmonary venous return [PAPVR] and sinus venosus atrial septal defect [SVASD]), surgically treated at the age of 3 years, associated with *Situs Viscerum Inversus* and polysplenia. She was promptly admitted to the Accident and Emergency Department of the Children’s Hospital of Palermo, Italy, with a 3-day history of left lower quadrant abdominal pain and vomiting. Over the past year, the patient had experienced similar episodes of left flank pain that resolved spontaneously. Family history was unremarkable, and both parents and two siblings were in good health. On admission, her temperature was 37.5 °C (99.5 °F), and the other vital signs showed normal values. Laboratory tests revealed leukocytosis (14,690 cells/µL, normal reference range [RR] 4-13.5 × 10^3^ cells/µL) with neutrophilia (78.4%, RR 40–60%), and increased C-reactive protein ([CRP] 13.03 mg/dL, RR < 0.5 mg/dL).

**Fig. 1:**
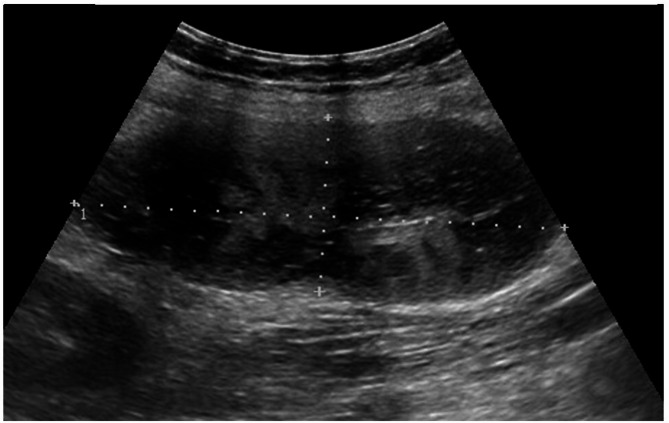
US scan shows a hypoechoic mass measuring 11 × 4 cm, with sharp margins, identified in the left flank - left iliac fossa, with no blood flow at Power-Doppler integration

On physical examination, the abdomen was tender and distended in the left lower. An abdominal ultrasound was performed, and identified a hypo-echoic mass measuring 11 × 4 cm, with sharp margins, in the left flank-left iliac fossa. Power-Doppler integration showed no blood flow in the mass (Fig. 1).

**Fig. 2:**
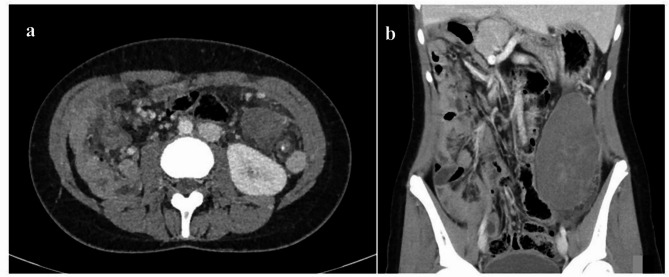
Axial (**a**) and coronal (**b**) CT scans confirm the presence of a mass and show a “whirlpool sign” involving the vascular pedicle of the twisted spleen, with a central “white dot sign”

A contrast-enhanced abdominal CT scan was subsequently obtained, and confirmed the presence of an 11 × 4 cm mass in the left flank-left iliac fossa, with sharp margins and poor-to-absent contrast enhancement. It showed a “whirlpool sign” involving the vascular pedicle of the twisted spleen, with a “white dot sign” already visible in the non-contrast scan, proximal to the area of torsion, due to the creation of an endoluminal thrombus in the afferent vessels (Fig. 2a**/b**). The CT scan also demonstrated other developmental abnormalities associated with polysplenia, some of which were already known from the patient’s medical history, including dextrocardia, midline liver, dorsal pancreatic agenesis, azygous continuation of the inferior vena cava and intestinal malrotation. In light of the clinical manifestations, in addition to the known condition of *Situs Viscerum Inversus* affecting the patient and the abnormal laboratory findings, a splenic torsion was suspected.

**Fig. 3:**
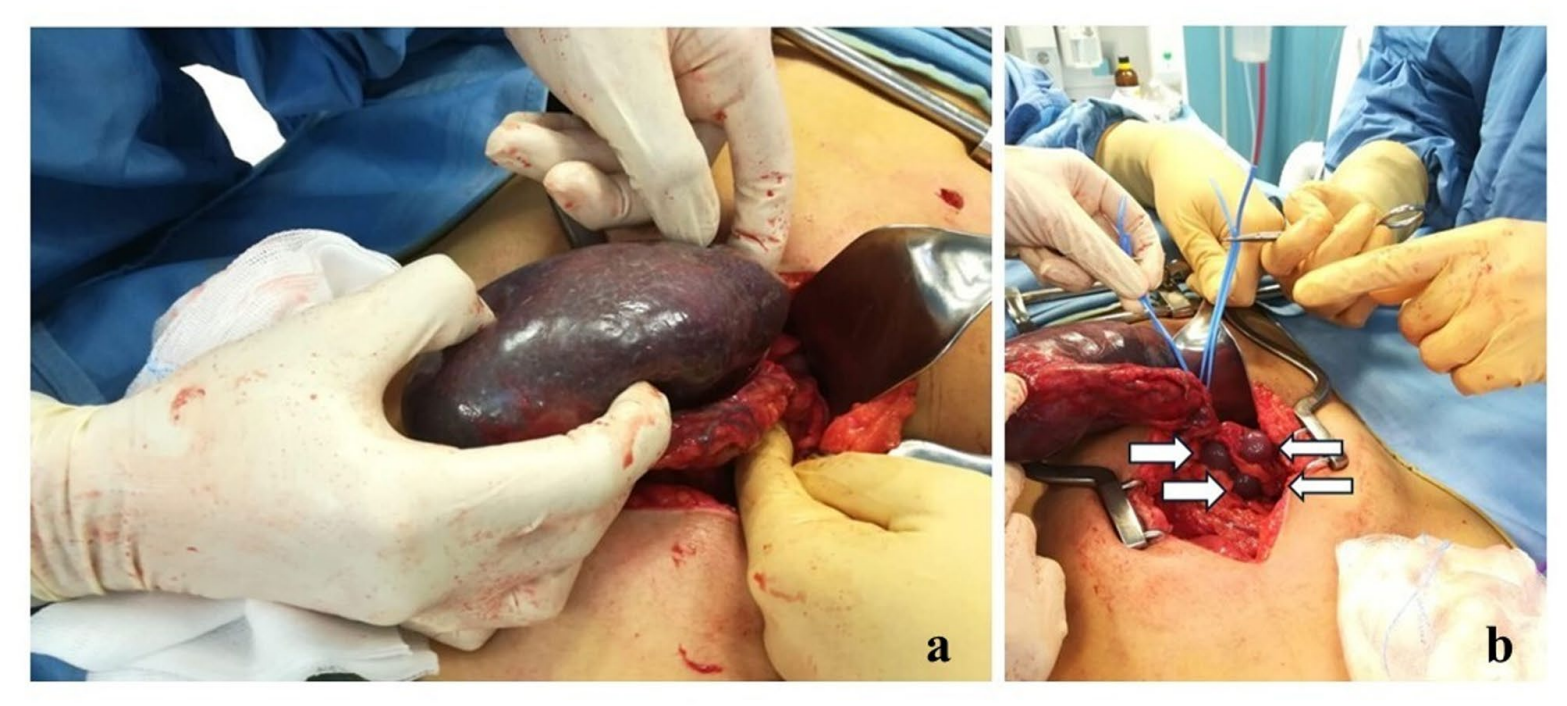
**a** Laparotomy confirms a pedunculated mass in the left flank - left iliac fossa, consistent with torsion of one of the spleens. **b** Four accessory spleens (indicated by arrows), each approximately 3 cm in size, were identified

The patient underwent urgent laparotomy, which confirmed a pedunculated mass (11 × 4) in the left flank-left iliac fossa, consistent with torsion of one of the spleens. The spleen appeared dark bluish-black in color, with an irregular surface, diffuse ecchymotic areas, and increased consistency. No splenic suspensory ligaments were present, consistent with a “wandering spleen.” There was complete torsion of the splenic pedicle, with two full rotations. Following detorsion, no restoration of normal vascular perfusion was observed; therefore, splenectomy was performed (Fig. 3a**/b**). Histological examination subsequently confirmed the diagnosis of splenic torsion. The postoperative course was uneventful, and she was discharged after 10 days.

## Discussion and conclusions

The management of children with acute abdomen represents a great diagnostic challenge in the emergency department, as the symptoms can be attributed to many diseases [17–22] (Table 1).

**Table 1:** Common causes of acute abdomen in children (divided by age groups)

Age Group	Common Causes of Acute Abdomen
Neonates (0–1 month)	- Volvulus - Hirschsprung’s disease - Necrotizing enterocolitis - Meconium peritonitis - Incarcerated hernia
Infants (1–12 months)	- Intussusception - Appendicitis (rare) - Volvulus - Incarcerated hernia - Acute gastroenteritis
Preschool (1–5 years)	- Acute appendicitis - Mesenteric adenitis - Gastroenteritis - Food poisoning - Volvulus or postsurgical adhesions
School-age (6–12 years)	- Acute appendicitis - Mesenteric adenitis - Severe constipation - Abdominal trauma - Inflammatory bowel disease
Adolescents (13–18 years)	- Acute appendicitis - Pelvic inflammatory disease - Ectopic pregnancy - Polycystic ovary syndrome (ovarian torsion) - Urolithiasis

Such complexity is even higher in cases of younger patients, due to their poor cooperation [20], or in the presence of associated anatomical anomalies, such as polysplenia. This latter may lead to splenic torsion, which requires thorough understanding and effective management. This condition indeed, although rare, can have serious consequences if not promptly recognized and treated, leading to severe prognosis and increased mortality risk. Its main causes are the lack of physiological fixation, as in cases of wandering spleen [21], or the presence of compression responsible for a mass effect on the vascular pedicle, secondary to anomalous position of the spleen. Actually, in cases of polysplenia, the chances of torsion can be increased due to the higher number of spleens and their poor fixation. Therefore, the occurrence of splenic torsion in polysplenia should always be considered in children with acute abdomen, even in the absence or presence of mild alterations of vital signs (e.g. fever) and laboratory test results (slight elevation of CRP and white blood cells), as occurred in the present patient. There are 15 reported cases of splenic torsion in pediatric patients with PSS. The presenting manifestations and associated alterations are variable and often not specific (Table 2). Gastrointestinal symptoms are commonly described, including abdominal pain and distension, vomiting, and fever [11, 16]. In rare cases, splenic torsion may also be asymptomatic [11].

**Table 2:** Reported cases of Splenic torsion in children and adolescents with PSS

	AGE/SEX	CLINICAL PRESENTATION	PHYSICAL EXAMINATION	LAB TEST	IMAGING					
					X-RAY	US DOPPLER	CT/MRI	OTHER	MANAGEMENT	EVOLUTION
1	13Y/F	ABDOMINAL PAIN, VOMITING	PAIN AT DEEPLY PALPATION IN RIGHT HYPOCONDRIUM	WBC +	X				LAPAROTOMY EXCISION	UNEVENTFUL, DISCHARGED AT 9 DAY POST SURGERY
2	3Y/F	ABDOMINAL PAIN, VOMITING	PALPABLE MOBILE MASS IN LLQ					TC-99 M SCINTIGRAPHY	LAPAROTOMY EXCISION	UNEVENTFUL
3	6Y/F	INTERMITTENT ABDOMINAL PAIN	PALPABLE MOBILE MASS IN EPIGASTRIUM		X	X			LAPAROTOMY EXCISION	UNEVENTFUL
4	8Y/F	3 DAYS ABDOMINAL PAIN IN RUQ AND BILIOUS VOMITING	ABDOMINAL TENDERNESS, PAIN AT DEEPLY PALPATION IN RIGHT HYPOCONDRIUM	WBC+, CRP+		X	X		LAPAROSCOPIC ESPLORATION + LAPAROTOMIC EXCISION	UNEVENTFUL, DISCHARGED AT 8 DAY POST SURGERY
5	7Y/F	ABDOMINAL PAIN	N/A			X		TC-99 M SCINTIGRAPHY	LAPAROSCOPIC EXPLORATION + LAPAROTOMY EXCISION	UNEVENTUFUL
6	9Y/F	RUQ ABDOMINAL PAIN	N/A				X		LAPAROSCOPY	UNEVENTFUL
7	17Y/F	LUQ ABDOMINAL PAIN	FEVER	WBC+, CRP+			X		CT FOLLOW UP (AT 3 MONTHS)	UNEVENTFUL
8	12Y/M	ACUTE ABDOMINAL PAIN, NON BILIOUS VOMITING	HIGH-GRADE FEVER WITH CHILLS AND RIGORS	WBC-, PLT-, PLASMODIUM VIVAX SCHIZONTS		X	X		LAPAROTOMY EXCISION	UNEVENTFUL, DISCHARGED AFTER 6 DAYS
9	10Y/F	2 DAYS HISTORY OF NAUSEA AND LEFT FLANK PAIN	FEVER (38.3). PALPABLE TENDER MASS IN THE LEFT FLANK.	WBC+		X	X		LAPAROTOMY EXCISION	UNEVENTFUL
10	15Y/F	ABDOMINAL PAIN ESPECIALLY IN RLQS	TENDERNESS ON PALPATION, DEFENSE AND REBOUND TENDERNESS AT THE RLQ	WBC+		X			LAPAROTOMY EXCISION	UNEVENTFUL, DISCHARGED AFTER 4 DAYS
11	9Y/F	3 DAYS ABDOMINAL PAIN IN THE LUQ, VOMITING, DIARRHEA, ANOREXIA	FEVER, TENDER MASS IN LUQ, AS WELL AS VOLUNTARY GUARDING,	WBC+, CRP+		X	X		LAPAROSCOPIC EXCISION	UNEVENTFUL, DISCHARGED AFTER 3 DAYS
12	13Y/F	ACUTE LOWER ABDOMINAL PAIN, NAUSEA AND FEVER.	TENDER LUMP IN THE RLQ	LDH ++		X	X		LAPAROTOMY EXCISION	UNEVENTFUL, DISCHARGED AFTER 4 DAYS
13	14Y/F	N/A	N/A	CRP+, WBC+		X	X		LAPAROTOMY EXCISION	UNEVENTFUL
14	8Y/M	INTERMITTENT DULL ABDOMINAL PAIN IN RUQ	TENDERNESS OVER THE RUQ REGION	WBC+, CRP+, D-DIMER+, HBG-			X	PET	LAPAROSCOPIC EXCISION	UNEVENTFUL, DISCHARGED AFTER 4 DAYS
15	3Y/M	ABDOMINAL PAIN IN RIGHT IPOCONDRIUM	FEVER	PLT+, WBC+		X	X		LAPAROSCOPIC EXCISION	UNEVENTFUL
16*	14Y/F*	3-DAY HISTORY OF LLQ ABDOMINAL PAIN AND VOMITING	ABDOMINAL TENDERNESS AND DISTENSION IN LLQ	FEVER, WBC+, CRP+		X	X		LAPAROTOMY EXCISION	UNEVENTFUL, DISCHARGED AFTER 10 DAYS

CRP: C-reactive protein; CE: contrast enhancement; HBG: hemoglobin; LDH: lactate dehydrogenase; LIF: left iliac fossa; LLQ: left lower quadrant; LUQ: left upper quadrant; N/A: not available; PET: positron emission tomography; PLT: platelets; RIF: right iliac fossa; RLQs: right lower quadrants; RUQ: right upper quadrant; WBC: white blood cells. *: our case

Ultrasound is often the first-line imaging investigation for evaluating patients with abdominal pain, although it may not be conclusive in all cases. However, radiological imaging plays an important role in the diagnosis of splenic torsion [23–25]. In this latter scenario, in fact, ultrasound with power-Doppler integration may show a hypoechoic, non-vascularized mass. Contrast medium CT and MRI, thereafter, can provide more detailed images. Actually, these scans may show a hypoattenuating and hypovascularized mass associated with a twisted vascular pedicle, as in our experience. Nonetheless, even these exams can also be inconclusive in some cases [16], leading to misleading findings. Imaging methods also allow for the detection of other anomalies associated with polysplenia, in patients with PSS where the condition is not previously known. This can raise clinical suspicion, guide diagnosis, and is important for preoperative planning. Most abdominal abnormalities can be identified through abdominal ultrasound, such as midline or left-sided liver, polysplenia, and pancreatic hypoplasia or abnormalities. Meanwhile, dextrocardia can be diagnosed through chest X-ray or echocardiography. Differential diagnosis for splenic torsion, however, is wide and complex, and includes wandering spleen, necrotic nodes, omental infarction, and chronic encapsulated fat necrosis. Currently, there are no established guidelines for its assessment in patients with polysplenia. Moreover, literature highlights significant heterogeneity in management approaches. In addition, the presence of many conditions from which it must be distinguished [26] can complicate the correct identification of the clinical picture. Then, it is crucial to have a clear understanding of the necessary investigations for patients with acute abdomen and known or suspected polysplenia (Table 1), to improve early diagnosis and optimize treatment.

Therapy of splenic torsion in patients with polysplenia is mainly surgical, with splenectomy being necessary in the majority of cases. In some subjects, such as those with wandering spleen, detorsion (untwisting the spleen) or conservative treatment may be attempted. This approach carries a higher risk of mortality, and splenectomy is still required if re-torsion occurs [15]. The choice of surgical approach is based on the degree and duration of torsion (partial or complete), the presence of splenic infarction or necrosis, and the lack of reperfusion following detorsion. In pediatric patients and young adults, preserving the spleen is often prioritized whenever feasible, due to its essential role in immune function. However, in patients with polysplenia syndrome, the resection of one of the spleens does not result in functional asplenia.

In the previously reported cases of literature (Table 2), there is a prevalence of splenic torsion in PSS among females. Nonspecific clinical manifestations (abdominal pain, non-biliary vomiting, fever), and laboratory test abnormalities (including leukocytosis and elevated CRP) were mainly reported. The most commonly used imaging techniques for diagnosis were Doppler ultrasound and CT. The management was surgical, with only one case treated conservatively, performing CT re-evaluation at 3 months. All patients undergoing surgery (laparoscopy/laparotomy) had an uneventful course.

Splenic torsion in Polysplenia Syndrome (PSS) is an extremely rare condition that may present in a time-critical manner, with nonspecific clinical manifestations and abnormal laboratory results, representing a real diagnostic challenge. When facing PSS children with acute abdominal pain, pediatricians should consider, among the possible causes, spleen torsion, which may require surgical intervention and/or careful monitoring.

## Data Availability

The datasets used and analyzed during the current study are available from the corresponding author on reasonable request.

## Abbreviations

CRP: C-reactive Protein
CT: Computed Tomography
MRI: Magnetic Resonance Imaging
PAPVR: Partial Anomalous Pulmonary Venous Return
PSS: Polysplenia Syndrome
RR: Reference Range
SVASD: Sinus Venosus Atrial Septal Defect
US: Ultrasound
